# Protein hydrolysates derived from poultry by-products as nutraceuticals: Bioactive potential, limitations, safety considerations, and commercialization perspectives

**DOI:** 10.1016/j.psj.2026.106956

**Published:** 2026-04-23

**Authors:** Yi-Hsieng S. Wu, Yi-Ling Lin, Yi-Chen Chen

**Affiliations:** a Department of Food Science, National Taiwan Ocean University, Keelung City 202301, Taiwan; b Department of Animal Science and Technology, National Taiwan University, Taipei City 106037, Taiwan; c Experimental Farm, College of Bioresources and Agriculture, National Taiwan University, Taipei City 106032, Taiwan

**Keywords:** Poultry by-products, Protein hydrolysates, Bioactive peptides, Food safety, Sustainable utilization

## Abstract

Over the past five decades, global agricultural production has increased substantially due to population growth, technological advancements, and farmland intensification. While this expansion has contributed to improved food security, it has also raised significant environmental concerns, driving international sustainability initiatives such as the United Nations 2030 Agenda and its 17 Sustainable Development Goals. In this context, circular economy strategies have gained attention for their potential to convert agricultural by-products into value-added resources, thereby reducing environmental burdens while creating economic opportunities. The poultry industry represents a major source of agricultural by-products, generating large quantities of feathers, blood, offal, and eggshells each year, underscoring the urgent need for sustainable utilization pathways. This review summarizes recent advances in the production of poultry by-product derived protein hydrolysates and evaluates their potential as bioactive nutraceuticals. Evidence regarding antioxidant, anti-inflammatory, and antimicrobial activities is critically examined, alongside key barriers to industrial application, including unclear mechanisms of action, batch-to-batch variability, consumer safety concerns, and regulatory constraints. Although poultry protein hydrolysates exhibit promising nutraceutical potential, further research is required to standardize production processes and address legal and safety challenges. Initial applications in animal and pet nutrition may provide practical models for safety assessment and regulatory approval, facilitating future translation into human nutrition. Interdisciplinary collaboration across food science, biotechnology, toxicology, and regulatory science will be essential to advance sustainable poultry by-product valorization.

## Introduction

Over the past five decades, global agricultural production has nearly tripled, largely driven by farmland expansion, the adoption of Green Revolution technologies, and rapid population growth. Although this surge in productivity has alleviated food shortages and improved living standards, it has simultaneously heightened environmental pressures, including soil degradation, water scarcity, biodiversity loss, and increased greenhouse gas emissions. These challenges underscore the urgent need to transition to resource-efficient production systems that balance the competing demands of food security, environmental preservation, and socio-economic development. Global initiatives such as the Millennium Development Goals (MDGs) and the 2030 Agenda for Sustainable Development (United Nations (UN), 2015a, United Nations (UN), 2015b; FAO, 2017) have provided a robust policy framework for governments and stakeholders to pursue this balance. Specifically, the 2030 Agenda, encompassing 17 Sustainable Development Goals (SDGs) and 169 specific targets, embodies a collective global commitment to promoting sustainability through responsible production, consumption, and technological innovation. In alignment with these global visions, countries worldwide have enacted green policies, reinforced environmental regulations, and developed monitoring systems to foster resource efficiency, circularity, and waste reduction, signaling a global shift toward sustainable agricultural and food systems.

Promoting a circular economy is a key strategy for sustainability, particularly by converting animal waste and agricultural by-products into valuable resources. Materials once regarded as disposal burdens are now recognized as feedstocks for energy, biochemicals, and functional ingredients (Toop et al., 2017; Sathish Kumar et al., 2024). The AgroCycle concept exemplifies this approach by repurposing agricultural wastes, by-products, and co-products through innovative technologies and practical business models (Toop et al., 2017). Agricultural waste includes food processing by-products, slaughterhouse residues, and farm-level discards, all of which present both environmental challenges and economic opportunities (Alayu and Yirgu, 2018; Limeneh et al., 2022). The rapid accumulation of agricultural waste represents both an environmental threat and an economic opportunity, prompting scientific and industrial interest in innovative valorization strategies (Sathish Kumar et al., 2024). The European Commission (2025) updated its circular economy monitoring framework in 2025 to strengthen sustainable resource use. Europe’s circularity rate is about 12% and is targeted to reach 24% by 2030, underscoring the importance of resource recirculation for environmental and climate goals. Notably, unprocessed fruit and vegetable waste is a major global issue, especially in China, the Philippines, India, and the United States. China generates about 32 million tons of organic waste, and the U.S. about 15 million tons, underscoring the need for circular strategies to convert organic waste into value-added products (Ravindran et al., 2022; Tuly et al., 2022; Mohanty et al., 2022).

The circular bioeconomy goes beyond waste reduction by using bioconversion technologies, such as enzymatic hydrolysis, fermentation, and anaerobic digestion, to convert organic residues into energy, fertilizers, and value-added bioproducts (Toop et al., 2017). As most agricultural by-products have low economic value, their valorization has become a priority. Recent advances in biotechnology and process engineering now enable the recovery of proteins, lipids, and bioactive compounds, supporting waste minimization and closed-loop production. The integration of these technologies has emerged as a focus for both industrial innovation and academic research, particularly in the livestock and poultry sectors, where the generation of edible by-products is massive and continuous. The poultry industry has grown rapidly, driven by rising demand for meat and eggs, making poultry by-products an increasingly important resource (Lasekan et al., 2013). Poultry meat now accounts for over 40% of global meat production, while egg production has risen from 15 to 93 million tons (FAO, 2025). The leading poultry producers, i.e., the United States, China, Brazil, and the EU, reported annual chicken meat production of 21.34, 15.35, 15.0, and 11.49 million metric tons, respectively, in 2024/2025 (USDA, 2025). However, this growth also generates massive amounts of waste and by-products, creating serious environmental concerns and highlighting the need to valorize poultry edible by-products for sustainable innovation (Agblevor et al., 2010; McGauran et al., 2021). For instance, in the UK alone, an estimated 1.1 billion birds are slaughtered each year, resulting in over 1 billion tons of waste and by-products, including bones, feathers, offal, and blood (Department for Environment, Food and Rural Affairs, 2020).

**Fig. 1:**
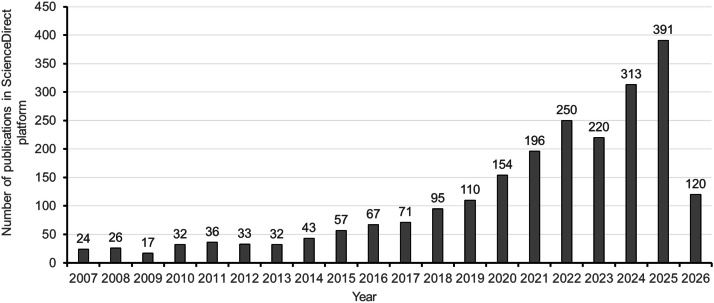
Number of research articles on “poultry by-product hydrolysates” published over the past 20 years. Data were retrieved from the ScienceDirect platform (https://www.sciencedirect.com/search?qs=poultry+by-product+hydrolysates) using the keyword “poultry by-product hydrolysates”, with the search period up to Feb. 8, 2026.

The health-promoting potential of food-derived hydrolysates or peptides has attracted considerable attention from food scientists in recent decades (Samaranayaka and Li-Chan, 2011). These functional hydrolysates or peptides, which are originally inactive within their parent proteins, can be released through enzymatic hydrolysis during gastrointestinal digestion, fermentation, ripening, or food processing. Kim and colleagues (2012) reported that protein hydrolysates exhibit beneficial bioactivities that are either as good as or better than those of the parent proteins. Moreover, undesirable components and off-flavors are often reduced or eliminated during hydrolysis, thereby improving sensory acceptability. Recent research has highlighted the potential of hydrolysates derived from poultry by-products as valuable bioactive materials (Fig. 1), with the number of publications related to the term “poultry by-product hydrolysate” on the ScienceDirect platform increasing from 12 to 376 over the past two decades, which reflects the growing academic and industrial interest in this field. Some short-chain peptides in these hydrolysates, produced through the hydrolysis of poultry offal, blood, feathers, and other by-products, exhibit notable biological activity, making them promising candidates for nutraceutical uses (Zou et al., 2021; Romero-Garay et al., 2021 & 2024; Bravo et al., 2023; Romero-Garay et al., 2024). Studies have shown that these hydrolysates possess antioxidant, anti-inflammatory, and antimicrobial properties, offering potential applications in health supplements, cosmetic formulations, and food preservation systems (Wu and Chen, 2022). In addition, specific fractions derived from poultry liver and muscle proteins have also exhibited additional biofunctional effects, such as glucose regulation, lipid metabolism modulation, and antihypertension, further broadening their application potential (Chen et al., 2017; Romero-Garay et al., 2021; Bravo et al., 2023; Lin et al., 2024a&b). Beyond reducing edible by-product waste, these applications align with the increasing global demand for natural, sustainable bioactive ingredients in the food and health industries. The valorization of poultry edible by-products through biotechnological processes thus represents an intersection between food technology, environmental science, and public health. This shift towards reusing poultry edible by-products reflects a broader agricultural trend to adopt circular economy practices. By transforming poultry byproducts into high-value products, the poultry industry can significantly reduce its environmental footprint while meeting consumer demand for sustainable ingredients.

Nevertheless, realizing the full potential of poultry edible by-product hydrolysates requires overcoming several significant challenges, including standardizing processing methods, ensuring product safety and efficacy, and complying with international regulations governing food-grade and pharmaceutical-grade materials. In addition, variability in raw material composition, enzymatic specificity, and process scalability remains a major technical constraint. Addressing these issues will require multidisciplinary collaboration among academia, industry, and regulatory bodies. Future efforts should focus on establishing comprehensive certification frameworks, developing pilot-scale biorefineries, and integrating risk assessment systems to ensure consistent quality. Ultimately, integrating circular bioeconomy principles into poultry production can serve as a model for sustainable development in other livestock industries, contributing to global goals for waste reduction, resource efficiency, and climate mitigation.

## Limitation of biofunctional mechanism determination

### Insufficient mechanistic characterization of poultry by-product hydrolysates

Understanding the mechanisms underlying biofunctional activities is foundational in both pharmaceutical and nutritional sciences, where rigorous scientific validation of specific effects on humans and other living organisms is paramount. Such scientific rigor underpins the credibility of health claims and the acceptance of bioactive ingredients in global regulatory frameworks. Accordingly, the clear identification of bioactive compounds, their molecular targets, and their pathways of action is critical for establishing efficacy and safety standards. However, research on the efficacy of hydrolysates from poultry by-products remains limited, and the precise characterization of their bioactive compound profiles is still insufficient. The variety of hydrolysates or biopeptides is influenced by the raw material source, enzyme hydrolysis methods, enzyme types, and other processing parameters, making it difficult to identify specific bioactive compounds and fully elucidate their mechanisms of action. Instead, many studies rely on amino acid composition data or dipeptide concentrations to infer effects (Onch et al., 2014, 2015, & 2016; Yang et al., 2014 & 2019; Nikolaev et al., 2016; Yusop et al., 2016; Nie et al., 2017; Bravo et al., 2019; Mas-Capdevila et al., 2018, 2019a & b, & 2020; Shang et al., 2026) (Table 1). While such studies provide preliminary insights, they often fall short of delivering the mechanistic precision required to establish causal links between specific peptide structures and observed physiological effects. As a result, many biological activities are vaguely attributed to “synergistic effects”, a term that lacks scientific rigor and does not meet the mechanistic standards expected in the fields of pharmaceuticals or nutritional science.

**Table 1:** Major research teams working on bioactive protein hydrolysates or peptides derived from poultry by-products or wastes.

Protein source	Extraction description	Feature description of hydrolysate/peptides	Model	Functionalities & rationales	References
Poultry viscera	[Autolytic degradation] A 40% (w/v) tissue homogenate was prepared, adjusted to pH 2.8, and incubated at 55°C for 6 h. The mixture was then centrifuged, neutralized, and dried into powder.	84.31±2.36 mg peptides per mL of hydrolysate; sequences: ARIYH, LRKGNLE, RVWCP	*In vitro*	[Anti-oxidation] DPPH, ABTS, superoxide & hydroxyl radical-scavenging activity, TEAC, reducing power, β-carotene bleaching assay	Jamdar and Harikumar (2008). Jamdar et al. (2012) Mane and Jamdar (2017)
			*In vitro*	[anti-hypertension] ACE inhibitory activity	
Turkey head	[Enzymatic hydrolysis] Turkey heads were homogenized in water (1:5 w/v), acidified to pH 2.5, and centrifuged to collect collagen. This was then re-homogenized (1:90 w/v), heated to 80°C, adjusted to pH 8, and diluted to 100 mL. After enzymatic hydrolysis at 50°C for 24 h, the mixture was reheated, centrifuged, and freeze-dried to yield collagen peptide powder.		*In vitro*	[Lipid-lowering effects] bile-acid binding ability	Khiari et al. (2014)
				[Anti-oxidation] POA inhibitory activity	
Duck liver	[Microbial hydrolysis] Duck livers were homogenized with water (ratios 2:1 to 6:1, v/v), autoclaved at 121°C for 15 min, cooled, and inoculated with *Bacillus subtilis* BNCC109047 (2–6%, v/v). Fermentation was conducted at 37°C (200 rpm) for 12–60 h. The fermented mixture was centrifuged (10,000×g, 4°C, 10 min), filtered (0.22 μm), lyophilized, and stored at -40°C as fermented duck liver extract.	< 1 kDa; sequences: MYGAVTPVK, NWEKIR, APGIIPR, and RWWQLR	*In vitro*	[Anti-oxidation] DPPH, ABTS, and hydroxyl radical-scavenging activity	Fan et al. (2023)
Duck gizzard	[Enzymatic hydrolysis] Duck gizzards (100 g) were stored at –20°C, then hydrolyzed with papain (0.2 g) under optimal conditions (pH 6.0, 50°C) for 3 hours. The reaction was terminated by boiling for 5 min, then cooled, filtered through ultrafiltration membranes (<3 kDa), lyophilized, and stored at -18°C.	<3kDa	*In vitro*	[Anti-oxidation] DPPH, superoxide and hydroxyl radical-scavenging activity [Anti-apoptosis] cell DNA damage assay	Su et al. (2016)
Duck skin	[Enzymatic hydrolysis] Duck skin gelatin was ground to powder and hydrolyzed under optimal enzymatic conditions (e.g., Alcalase at pH 8.0, 50°C). For hydrolysis, 1 L of phosphate buffer and 0.2 mL of enzyme were added per kilogram of sample, and the reaction was maintained for 8 h. The hydrolysis was terminated by heating at 100°C for 10 min, followed by cooling. The resulting hydrolysates were freeze-dried for 3 days to obtain gelatin hydrolysate powder.	WYPAAP, and MW is 693.90 Da	*In vitro*	[Anti-oxidation] DPPH, Alkyl, superoxide, and hydroxyl radical-scavenging activity	Lee et al. (2012) Lee et al. (2013)
				[Anti-apoptosis] cell apoptosis assay, western blotting for Bax and Bcl-2 proteins	
Chicken liver	[Enzymatic hydrolysis] Chicken livers were blended with distilled water at a ratio of 1:2 (w/v), adjusted to pH 2.0, and heated to 95°C. Pepsin was added at a ratio of 1:400 for 2 h at 37°C to achieve optimal hydrolysis. The reaction was terminated by heating, followed by centrifugation, filtration, and freeze-drying. The resulting hydrolysates were used for yield determination, peptide content analysis, and antioxidant activity assessment.	< 10 kDa, total amino acid composition, carnosine/anserine, and mineral contents	*In vitro*	[Anti-oxidation] DPPH radical-scavenging activity, ferrous ion chelating ability assay, D-galactose induced mice model	Chou et al. (2014). Yang et al. (2014) Lin et al. (2017, 2023, & 2024a&b) Chen et al. (2017) Wu et al. (2020 & 2021) Yeh et al. (2022)
			*In vitro*	[Anti-obesity] pancreatic lipase activity assay and bile-acid binding ability in high-fat diet-induced hamster model	
			*In vivo*	[Hepatoprotection] Chronic alcoholic diet-induced mice model, alcohol-fed rat model, thioacetamide-induced rat model, and high-fat diet-induced mice model	
			*In vivo*	[Cardio-renal protection] high-fat diet-induced mice model	
			*In vivo*	[Anti-cognitive decline] streptozotocin-induced diabetic mice model	
			*In vitro* *In vivo*	[Anti-diabetes] FL83B and C2C12 cell model, db/db mice model	
Chicken liver	[Enzymatic hydrolysis] Chicken livers were minced, heated at 100°C for 20 min, and treated with 10% isopropanol (1:10, w/v) for 12 h. After centrifugation (10,000 rpm, 4°C), the defatted liver was mixed with NaOH (pH 8.0, 1:6, w/v) at 50°C and hydrolyzed with trypsin (4,000 U/g). The reaction was terminated by heating at 100°C for 20 min, followed by centrifugation (5,000 rpm, 4°C, 15 min) and lyophilization of the supernatant.		*In vitro*	[Anti-oxidation] DPPH and hydroxyl radical-scavenging activity	Xiong et al. (2020)
Chicken liver	[Enzymatic hydrolysis] Chicken-liver protein hydrolysate was prepared by inactivating homogenates at 100°C for 10 min, then hydrolyzing with six enzymes (1% w/w) for 3 h under optimal conditions. After terminating the reaction at 100°C, the mixture was centrifuged, and the supernatant was freeze-dried and stored at 4°C.	Sequences: WYR, MMR, KPFPA, and ALLPL	*In vivo* *In vitro*	[Anti-aging] *C. elegant* model	Chen et al. (2024a)
				[Anti-oxidation] DPPH, ABTS, and hydroxyl radical-scavenging activity	
Chicken skin	[Enzymatic hydrolysis] Fresh chicken skins were freeze-dried, shredded, defatted with acetone, air-dried, and milled into powder, then stored at -20°C. For enzymatic hydrolysis, 5% slurries of the powder were prepared. Alcalase was added to pH 8.0 slurries at 55°C for 4 h, while pepsin+pancreatin was used at pH 2.0 and 37°C, followed by pH 7.5 with pancreatin at 37°C for 4 h. Both reactions were terminated by heating to 95°C, centrifuged, and freeze-dried.	Total amino acid profile	*In vitro*	[Anti-oxidation] DPPH, superoxide, and hydroxyl radical-scavenging activity, chelation of metal ions assay, oxygen radical absorbance capacity	Onuh et al. (2014, 2015, & 2016)
			*In vitro/In vitro*	[Anti-hypertension] ACE and renin inhibitory activity and the spontaneously hypertensive rat (SHR) model	
Chicken feather	[Microbial hydrolysis] Using *Chryseobacterium sp. kr6* for feather hydrolysates, optimal soluble protein (18.5–22 mg/mL) was achieved with 50–75 g feathers/L/L at pH 6.0–9.0 after 48 h. Protease production peaked at 24 h regardless of feather concentration or pH, and maximum antioxidant activity was observed with 50 g feathers/L/L at pH 8.0 after 48 h at 30°C.	<10kDa	*In vitro*	[Anti-oxidation] DPPH and ABTS -scavenging activity	Fontoura et al. (2014)
				[Anti-hypertension] ACE and DPP-IV inhibitory activity	
				[Anti-apoptosis] cell DNA damage assay	
Chicken neck	[Enzymatic hydrolysis] Broiler necks were processed to produce a functional animal protein product (FAPP) through a series of steps. The necks were first ground and adjusted for moisture content, then subjected to enzymatic hydrolysis. The reaction was terminated by heating at 93°C for 30 min. The hydrolysate was then defatted, refined, and sterilized at 120°C for 20 min. Finally, the material was concentrated and spray-dried to obtain the FAPP.	Free amino acid contents	*In vitro*	[Anti-oxidation] TEAC assay and ORAC-FL assay	Nikolaev et al. (2016)
Chicken skin	[Enzymatic hydrolysis] Broiler and spent hen skins were extracted in 1 M NaCl, defatted with acetone, and treated with 0.1 N NaOH at 100°C. Insoluble elastin was solubilized in oxalic acid. The isolated elastin (61% and 67% protein, respectively) was hydrolyzed using Alcalase at 60°C and pH 8.5, or Elastase at 37°C and pH 8.5, for 2–24 h. Following enzymatic inactivation, the hydrolysates were centrifuged and lyophilized into peptide powders, which were stored at −18°C until use.	Total amino acid composition	*In vitro*	[Anti-hypertension] ACE inhibitory activity	Yusop et al. (2016)
Chicken bone	[Enzymatic hydrolysis] Chicken bones were cooked in water (1:1.5, w/v) at 130°C for 20 min, then ground into a slurry. The slurry was hydrolyzed with a mixed enzyme blend at 55°C for 3.5 hours, heated to 100°C to stop the reaction, and centrifuged to remove fat and residual material. The hydrolysate (CBPH) was separated using ultrafiltration into two fractions, CBPH-I (<3 kDa) and CBPH-II (3–10 kDa), then lyophilized and stored at 4°C.	Free amino group contents	*In vitro*	[Anti-oxidation] DPPH and hydroxyl radical-scavenging activity, reducing power	Nie et al. (2017)
Chicken foot	[Enzymatic hydrolysis] Chicken feet were processed to obtain Hpp11 hydrolysate. Protein powder (20 mg/mL) was preheated at 100°C for 1.5 h, then hydrolyzed with Protamex® (2.67 µg/mL) at 50°C and pH 7.0 for 2 h. The enzymatic reaction was terminated by heating at 80°C for 10 min. The hydrolysate was centrifuged, filtered, and freeze-dried for subsequent use.	Total nitrogen compounds content and free a-amino group content; sequences: AVFQHNCQE and QVGPLIGRYCG	*In vivo*	[Anti-hypertension] ACE and renin inhibitory activity, the spontaneously hypertensive rat (SHR) model	Bravo et al. (2019) Mas-Capdevila et al. (2018, 2019a&b, & 2020)
Chicken blood	[Enzymatic hydrolysis] Boiled chicken blood was blended, and 1 g of homogenate was diluted with 2 mL of distilled water (500 mg/mL) and heated at 80°C for 15 min. A two-stage enzymatic hydrolysis was performed, followed by enzyme inactivation at 90°C for 10 min. Hydrolysates were stored at -20°C, with all samples prepared in triplicate.	>10 kDa,	*In vitro*	[Anti-obesity] bile-acid binding ability	Carrera-Alvarado et al. (2023)
Chicken egg chalaza	[Enzymatic hydrolysis] Hydrolysis was conducted with pepsin (pH 2, 37°C), protease A (pH 6, 50°C), or prozyme 6 (pH 8, 45°C) at a 1:200 (w/w) enzyme-to-substrate ratio for 0–4 h. The optimal enzyme and conditions were identified and optimized using enzyme-to-substrate ratios of 1:100–1:500 (w/w). Hydrolysates were heat-inactivated (95°C, 15 min), centrifuged (900 × g, 4°C, 15 min), filtered, and lyophilized.	< 15 kDa, free amino acid composition, and carnosine/anserine contents	*In vitro*	[Anti-oxidation] DPPH and ABTS^+^ radical-scavenging activity, Reducing power	Yang et al. (2019) Chan et al. (2020) Chen et al. (2020; 2021) Lin et al. (2021)
			*In vivo*	[Hepatoprotection] chronic alcohol consumption-induced liver steatosis mouse model; high-fat diet-induced hamster model; high-fat diet-induced rat model	
			*In vivo*	[Anti-cognitive decline] D-galactose-induced mouse model	
			*In vivo*	[Anti-obesity] high-fat diet-induced hamster model	
Chicken eggshell membrane	[Enzymatic hydrolysis] Eggshell membrane hydrolysate was prepared by dispersing 5 g of ESM powder in 100 mL buffer (pH 8.0) with Na_2_SO_3_. After adding alkaline protease (9 U/mg), the mixture was incubated at 55°C for 4 h. The enzyme was inactivated at 80°C for 30 min. The solution was centrifuged, dialyzed, and freeze-dried to obtain ESMH powder.	31 kDa	*In vivo*	[Wound healing] wound healing mouse model	Wang et al. (2024)
Duck egg-white	[Enzymatic hydrolysis] Boiled duck-egg white was mixed with an equal volume of ddH_2_O, followed by homogenization. The hydrolysis reaction was carried out with protease A (63,000 U/g; Amano Enzyme Ltd., Nagoya, Japan) at a 1:100 (w:w) ratio (enzyme to boiled duck-egg white) at pH 6.0 and 50°C for 2 h. The hydrolysate was subjected to freeze-drying.	Total amino acid composition and carnosine/anserine	*In vivo* *In vitro*	[Muscle-endurance improvement and anti-fatigue activities] High-intensity exercise in mice and dexamethasone-induced C2Cl2 cell model	Shang et al. (2026)

### Bottlenecks: Limitations in peptide sequencing and computational prediction tools, and current *in vitro* screening models

To address these limitations, two primary research bottlenecks must be overcome. The first involves developing rapid, precise peptide sequencing platforms and analytical tools. Recent technological advancements in proteomics have led to improved tools for peptide sequencing and functionality prediction (Petrovskiy et al., 2024; Yilmaz et al., 2024). High-resolution tandem mass spectrometry (MS/MS) coupled with bioinformatics databases has greatly enhanced the accuracy of peptide identification from complex hydrolysate matrices (Lv et al., 2017; Xie and Butler, 2025; Sun et al., 2025). When integrated with machine learning-based activity prediction models, these technologies can accelerate the discovery of functional peptides and help predict their biological interactions. Artificial intelligence (AI) and deep-learning algorithms are increasingly applied to simulate receptor-ligand interactions and molecular docking behaviors, offering a novel approach to mapping structure-activity relationships and predicting potential biofunctional roles (Chen et al., 2024b). Despite these advances, translating computational predictions into experimentally validated evidence remains a major challenge, largely due to the limited availability of high-quality, food-derived peptide datasets for model training. Expanding peptide databases with standardized annotation, verified activity, and toxicity profiles will be a key step in improving AI model reliability and generalizability across peptide types. Furthermore, establishing open-access repositories that integrate peptide sequence data with physicochemical parameters, digestion stability, and bioactivity records could foster cross-disciplinary research and enhance data transparency for reproducibility and meta-analyses.

The second bottleneck pertains to the development of efficient and representative *in vitro* screening platforms. To date, studies that successfully identify and validate active peptide sequences from protein hydrolysates have primarily focused on antioxidant and antihypertensive properties, due to the availability of reliable *in vitro* screening systems for these effects, such as angiotensin-converting enzyme (ACE) inhibition assay, dipeptidyl peptidase 4 (DPP4) inhibition assay, 1,1-diphenyl-2-picrylhydrazyl (DPPH) radical scavenging assay, reactive oxygen species (ROS) assay, etc. (Romero-Garay et al., 2021; Bravo et al., 2023). These methods are reliable for small peptides (typically under 25 amino acids) that can be easily synthesized, purified, and analyzed. However, more complex physiological effects, such as anti-fatty liver, hepatoprotective, lipid-lowering, anti-obesity, blood sugar-regulating, muscle-endurance-improving, and anti-fatigue actions, depend on multi-organ systems and complex metabolic pathways, making them challenging to replicate with current *in vitro* models (Table 1). Thus, simple biochemical assays are insufficient for evaluating such multifactorial biological effects.

### Integrating simplified cell models and *in vivo* studies

Several studies have attempted to bridge this gap by employing simplified cell-based models to simulate key metabolic characteristics, followed by validation in animal experiments (Wu et al., 2021; Lin et al., 2024a). For example, Wu and colleagues (2021) observed that their developed chicken-liver hydrolysate effectively attenuates lipid accumulation induced by 400 μM oleic acid (OA) in hepatocyte-derived FL83B cells. A similar observation was also demonstrated in hepatic lipid deposition in mice fed a long-term high-fat diet (20 weeks), and the underlying modulatory mechanisms were further verified in 400 μM OA-treated FL83B cells by the same research team. In follow-up studies, Lin and colleagues (2024a) reported that supplementation with chicken-liver hydrolysate could enhance glucose uptake. They upregulated the expressions of insulin receptor β (IRβ), phosphorylated Akt to total Akt ratio (p-Akt/Akt), and phosphorylated glycogen synthase kinase-3 to total GSK-3 ratio (p-GSK3/GSK3) in tumor necrosis factor-α (TNF-α)-induced FL83B hepatocytes and C2C12 myotubes. Furthermore, using db/db mice with a leptin receptor mutation, a well-established model of type II diabetes, they confirmed that administration of chicken liver hydrolysate ameliorated insulin resistance and improved metabolic homeostasis. Additionally, more than 80% of duck eggs are processed into traditional preserved products, such as century eggs (pidan) and salted eggs. A processing technique that separates and marinates duck-egg yolks directly has recently been developed to improve production efficiency. However, the remaining duck-egg whites often become underutilized by-products due to poor consumer acceptance, limited applications, and low storage stability. Shang and colleagues (2026) developed a functional duck-egg white hydrolysate (DEWH) that contains higher levels of BCAAs and imidazole dipeptides (carnosine and anserine) compared with Brand’s chicken essence. Using a high-intensity exercise mouse model, their study demonstrated that DEWH supplementation could enhance muscle endurance, as evidenced by increased hindlimb muscle mass, enlarged soleus fiber size, improved swimming and treadmill performance, reduced exercise-induced markers of muscle injury and inflammatory cytokines, and enhanced anti-fatigue capacity through accelerated lactate clearance. Furthermore, the molecular mechanism of DEWHs was investigated using dexamethasone-induced C2C12 cells, showing that DEWHs promote protein synthesis (upregulation of p-p70S6K/p70S6K and p-4EBP1/4EBP1; downregulation of p-FoxO1/FoxO1 and TRIM63) and reduce ATP depletion by enhancing mitochondrial biogenesis factors (NRF1 and PGC-1α). Although these integrated cell models and *in vivo* approaches provide valuable mechanistic insights and demonstrate potential therapeutic relevance, they still fall short of establishing an ideal, rapid screening platform for identifying active hydrolysates or peptides and their biofunctionalities. Major unresolved issues, including peptide stability, structural modification, intestinal absorption, and *in vivo* bioavailability, continue to hinder the efficient identification and validation of functional peptides.

### Multi-omics approaches to mechanistic elucidation

Beyond traditional biochemical assays, multi-omics integration combining transcriptomics, proteomics, and metabolomics could offer deeper insight into peptide-target interactions and systemic responses. By capturing the cascade of molecular changes induced by peptide exposure, multi-omics provides a comprehensive view of biofunctionality, linking molecular signatures to phenotypic outcomes. For instance, transcriptomic profiling can identify gene networks responsive to specific peptide treatments, while metabolomic analysis reveals downstream biochemical shifts reflective of metabolic regulation. When combined with systems-biology modeling, these datasets can elucidate cause-and-effect relationships, improving understanding of how peptides influence cellular and physiological functions. However, these approaches require advanced computational infrastructure, data harmonization, and robust statistical modeling pipelines that remain under development. Furthermore, interspecies variability between animal or cell models and human physiology poses an additional challenge, often leading to inconsistent efficacy data in translational research. To bridge this gap, developing human organoid or microphysiological models that replicate human tissue responses to bioactive peptides could significantly improve translational accuracy (Steinway et al., 2020; Lai et al., 2022).

### Challenges in peptide absorption, stability, and bioavailability

**Fig. 2:**
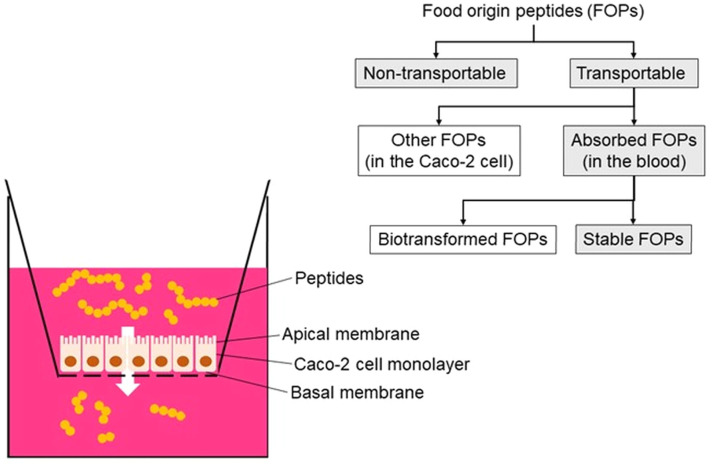
Classification of food-origin peptides (FOPs) during simulated intestinal absorption via Caco-2 monolayer model (modified from Liu et al., 2024). The yellow polymer in the cell medium indicates the peptides. The grey text boxes highlight the major FOPs identified in this system's results.

**Fig. 3:**
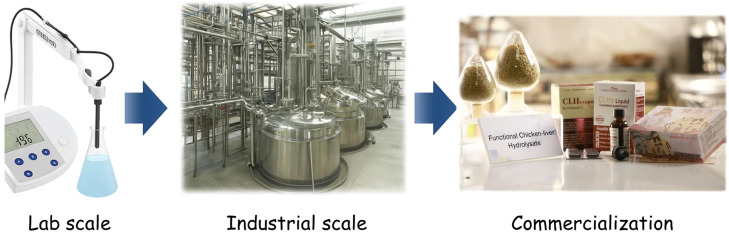
Process flow from lab-scale to industrial-scale production and commercialization of functional poultry by-product hydrolysates.

Peptide absorption and bioavailability introduce additional complexity to understanding their mechanisms of action. Although active peptides are susceptible to enzymatic or microbial degradation, numerous studies have shown that food-derived peptides can be absorbed through the intestine and enter the bloodstream, showcasing their potential bioactivity *in vivo* (Romero-Garay et al., 2021). Extensive research has elucidated protein metabolism and absorption pathways, identifying key transport proteins, such as peptide transporter 1 (PEPT1) and sodium-coupled oligopeptide transporter 1 (SOPT1)/SOPT2, within the brush border membrane, which facilitate peptide transport beyond passive diffusion and transcytosis (Ganapathy, 2012; Yu et al., 2024). Moreover, recent studies have highlighted paracellular transport as a significant pathway for peptide absorption, broadening the understanding of bioactive peptide transport mechanisms (Yu et al., 2024). Liu and colleagues (2024) further proposed a classification system for food-derived oligopeptides (FOPs) into 4 types: non-transportable, transportable, absorbed, and stable FOPs. This classification underscores the complexity of peptide absorption, as demonstrated using a stimulated epithelial absorption model in Caco-2 monolayers (Fig. 2). These findings emphasize the importance of understanding not only peptide function but also their transport kinetics and metabolic fate within the human body. Moreover, the role of enzymatic cleavage and microbial conversion in modifying peptide bioactivity and enhancing bioavailability adds another layer of complexity. These transformative processes suggest that food processing could significantly impact the efficacy of bioactive peptides, warranting further investigation into their mechanisms and implications (Liu et al., 2024). Consequently, understanding how processing parameters, such as pH, temperature, enzyme type, and hydrolysis duration, affect peptide formation, degradation, and release dynamics is essential for designing hydrolysates with predictable functional outcomes.(Fig. 3).

### Processing technologies and strategies to enhance bioavailability

In research focused on bioactive peptides derived from animal by-products, precise identification of functional peptide components is essential for elucidating their mechanisms of action and absorption pathways. Such mechanistic insight provides a scientific basis for applying optimized or emerging processing technologies to enhance peptide stability, bioavailability, and physiological efficacy. For instance, encapsulation strategies, such as liposome delivery systems or enzymatic inhibition approaches that prevent premature gastrointestinal degradation, can substantially improve peptide survival and absorption (Berraquero-García et al., 2023; Chen et al., 2025; Rahim et al., 2025). Gong et al. (2025) demonstrated that liposomal encapsulation confers robust protection against enzymatic hydrolysis for casein-derived peptides (CPs), enabling controlled intestinal release, promoting interactions with the gut microbiota, supporting nutrient uptake, and modulating gastrointestinal health. Additionally, integrating *in silico* peptide design with targeted enzymatic hydrolysis may facilitate the production of peptide sequences with predictable and customizable bioactivities (Hajfathalian et al., 2025; Liu, 2025a). Advancing these technological developments requires substantial foundational research to clarify peptide stability, transport mechanisms, and functional outcomes within biological systems. For example, Liu et al. (2025b) developed a β-glucan-functionalized liposomal delivery system to improve the oral efficacy of egg-white derived ACE inhibitory peptides (RADHPFL and YAEERYPIL). The delivery platform enhanced peptide stability, intestinal uptake, and antihypertensive activity, highlighting the potential of egg white peptides and targeted liposomal systems for managing hypertension.

At present, only a limited number of studies on bioavailability have examined bioactive peptides derived from poultry proteins. Research specifically addressing bioactive peptides from poultry by-products is even more scarce, indicating considerable potential for future development in this field. To fully realize the therapeutic and nutritional potential of peptides derived from underutilized edible poultry by-products, the field must prioritize systematic investigations that address existing mechanistic and technological gaps. Overcoming current bottlenecks in peptide identification, stability optimization, and absorption characterization will not only enhance scientific clarity for regulatory evaluation but also support the sustainable valorization of poultry-based by-products and broaden their applicability in functional food and nutraceutical development.

## Limitations in quality control and safety considerations

### Challenges in slaughterhouse design, by-product collection, and quality consistency

Poultry by-products, regarded as valuable protein sources for the production of bioactive hydrolysates, are primarily collected from slaughterhouses. To maximize their potential for reuse and value addition, the design and operation of poultry slaughterhouses must prioritize the efficient, hygienic, and traceable collection of by-products, guided by principles of sustainability and a circular bioeconomy (Kanani et al., 2020). Despite their high nutrient-rich content, maintaining consistent quality control of these raw materials presents a significant challenge. Historically, the architectural and operational designs of slaughterhouses were developed primarily to optimize waste removal and disposal efficiency, often combining offal, blood, and wastewater for solid–liquid separation. While effective for waste management, this traditional approach has inadvertently hindered the development of by-product valorization processes because blending waste streams introduces contamination risks that compromise material quality and safety.

In recent years, growing awareness of circular agriculture and environmental stewardship has shifted the industry’s focus toward sustainable utilization of by-products. However, integrating by-product reuse into existing slaughterhouse systems has introduced new challenges. The blending of waste streams increases the risk of microbial and chemical contamination, complicating compliance with increasingly stringent food safety standards (Kanani et al., 2020). Therefore, redesigning slaughterhouse workflows to separate and handle specific by-product categories under controlled conditions has become essential for enabling their safe transformation into value-added products such as protein hydrolysates. Controlled collection of liver, muscle, skin, fat, feathers, and blood under cold-chain conditions, coupled with immediate segregation and hygienic handling, is critical for reducing microbial load, maintaining nutrient integrity, and preventing cross-contamination. In addition to environmental monitoring, implementing automated sorting, conveyor, and cleaning systems can further reduce human contact, standardize raw material quality, and ensure operational efficiency. Training personnel in standardized hygiene protocols and monitoring environmental parameters such as temperature, humidity, and surface sanitation can further improve raw material quality.

### Uneven research attention and limitations across different by-product types

Among poultry edible by-products, the liver tissue has received the most extensive research attention for hydrolysate production. Chicken-liver derived hydrolysates have attracted considerable interest due to their relatively stable and safe raw material supply, particularly in regions where chicken liver remains a common food ingredient (Table 1) (Chou et al., 2014; Chen et al., 2017 & Chen et al., 2024b; Yang et al., 2014; Lin et al., 2017, 2023, & 2024a&b; Wu et al., 2020 & 2024; Xiong et al., 2020; Romero-Garay et al., 2021; Zou et al., 2021; Yeh et al., 2022). These hydrolysates are rich in peptides with antioxidant, antihypertensive, and hepatoprotective functions, making them promising candidates for functional food development. Nevertheless, expanding the scope of hydrolysate production to include other poultry tissues, such as muscle trimmings, skin, fat, feathers, and blood, requires substantial improvements in raw-material handling and waste-collection procedures to mitigate microbial and chemical contamination. Achieving consistent product quality and safety throughout the supply chain will be crucial for establishing consumer and regulatory confidence in poultry-derived hydrolysates (Kanani et al., 2020).(Table 2).

**Table 2:** Overview of the utilization pathways of chicken by-products and their respective legal categories defined under EU regulation (European Union, 2009).

Uses of chicken by-products	EU classification of the by-products
Livestock feed	Category 3
Pet food	Category 3
Aqua feed	Category 3
Cosmetic products	Category 3
Compost	Category 2 and 3
Production of biogas	Category 2 and 3
Production of thermal and electrical energy	Category 1, 2, and 3
Production of biofuel	Category 1, 2, and 3

### Microbial contamination risks and safety challenges in underutilized by-products

Different types of by-products face varying contamination risks, with microbial contamination the most prevalent concern. As we know, intestinal contents, improper evisceration, and delayed chilling can significantly increase bacterial loads. Traditionally, poultry by-products were primarily repurposed as nutrient additives in animal feed, a practice that remains a major area of research. By-products such as feathers, fat, skin, and blood remain valuable protein sources for animal nutrition (Martínez-Alvarez et al., 2015). However, their use presents challenges, including complex compositions, costly purification processes, and inconsistent quality and safety profiles, which can pose risks for animal consumption (Lecrenier et al., 2020). Further complicating this issue is the global restriction on the use of by-products from the same species due to concerns about infectious diseases. Studies have repeatedly highlighted microbial contamination, often originating from intestinal contents, as a common issue, emphasizing the importance of stringent quality control measures (Martínez-Álvarez et al., 2015; Stiborova et al., 2019). Without advancements in by-product collection and processing methods, the full spectrum of potential hazards and risks associated with these by-products remains poorly understood, posing challenges for regulatory agencies tasked with establishing safety guidelines.

### Chemical contaminants, processing-induced risks, and analytical requirements

The safety impact of atypical processing techniques in hydrolysate production warrants further investigation. For example, concentration and freeze-drying processes may inadvertently raise the levels of harmful substances. In chicken-liver-hydrolysate production, components such as animal drugs, antibiotics, and heavy metals, which may accumulate in the liver, must be thoroughly analyzed in the final product, especially after concentration (Chou et al., 2014; Zou et al., 2021). For example, studies by Chou and colleagues (2014) used government-certified chicken livers as raw materials to develop antioxidative chicken-liver hydrolysates. Subsequently, they examined the heavy metal content of the hydrolysates to address safety concerns arising from drying-induced concentration effects. Similarly, Yang and colleagues (2019) assessed antibiotic and sulfonamide residues in the chicken-egg chalaza before developing functional egg chalaza hydrolysates. Such safety operations should be incorporated at both laboratory-scale production and during scale-up for commercialization. Consequently, comprehensive quality evaluation of both raw materials and final products should include monitoring of spoilage bacteria, mycotoxins, heavy metals, and lipid peroxidation indices. Establishing robust analytical and control frameworks is therefore indispensable for ensuring the safety, consistency, and reliability of hydrolysate-based ingredients intended for functional food or nutraceutical applications.

### Storage stability, regulatory gaps, consumer perception, and future directions

Beyond microbiological and chemical hazards, oxidation and degradation processes during storage represent another quality concern. Exposure to oxygen, light, and heat may accelerate the oxidation of proteins and lipids, leading to the formation of aldehydes and other secondary oxidation products that reduce nutritional quality and generate off-flavors. Lipid oxidation is a primary cause of quality deterioration in poultry and poultry by-products, largely because poultry meat is rich in polyunsaturated fatty acids (PUFAs) and therefore highly prone to oxidative degradation (Domínguez et al., 2019). Besides, post-slaughter microbial safety is another key challenge for poultry meat and its by-products. Pathogens such as *Salmonella* and *Campylobacter* can contaminate carcasses during slaughter, and by-products often have higher microbial loads because they are typically processed later than primary meat cuts (Abdullah et al., 2025). Maintaining an unbroken cold chain, using oxygen-barrier packaging, and adding natural antioxidants during processing can mitigate these risks and extend shelf life. Furthermore, integrating hazard analysis and critical control points (HACCP) principles throughout all stages of by-product processing should be reinforced to identify, monitor, and control safety hazards systematically. Incorporating these preventive measures ensures that hydrolysates retain functional properties and meet the stringent standards required for human consumption.

Compared with intact proteins, hydrolyzed peptides generally exhibit higher bioactivity and digestibility. However, they are more sensitive to storage-related factors such as temperature, water activity, and oxygen, which can compromise their functionality. Consequently, stricter storage conditions are required to maintain their bioactivity and value (Romero-Garay et al., 2021). While regulatory authorities worldwide have established standards for managing animal by-products, harmonized global frameworks specifically governing food-grade poultry by-product hydrolysates remain lacking. Hydrolysates intended for human applications often fall into regulatory gray zones. The absence of universally accepted analytical methodologies for assessing hydrolysate safety further complicates international trade and product registration. Establishing standardized regulatory definitions, testing criteria, and labeling protocols is essential for promoting transparency and facilitating the global commercialization of poultry by-product hydrolysates. The adoption of advanced monitoring and digital technologies offers a promising pathway to enhance quality control. For example, blockchain-based traceability systems can record every step of by-product collection and processing, providing immutable verification of hygiene compliance and processing conditions (Sri Vigna Hema and Manickavasagan, 2024). Similarly, rapid biosensors and molecular detection kits can allow real-time monitoring of microbial or chemical contaminants at critical control points (Dong et al., 2023). These technologies, when integrated into smart manufacturing systems, can significantly improve process reliability, reduce human error, and strengthen consumer trust in hydrolysate-based products.

Public perception also plays a decisive role in the sustainable utilization of poultry by-products. As hydrolysates derived from animal sources are increasingly incorporated into functional foods, dietary supplements, and cosmetic formulations, consumer acceptance depends on transparent communication about safety assurance and environmental benefits. Labeling initiatives and third-party certification, such as “upcycled ingredient”, “sustainably sourced”, or “traceable origin”, can serve as effective tools to promote confidence and differentiate high-quality products. Educational campaigns emphasizing the nutritional and environmental advantages of converting by-products into functional ingredients may further help reshape consumer attitudes, moving them from viewing by-products as “by-product reuse” to recognizing them as “resource valorization”. Ultimately, achieving both safety assurance and quality consistency in poultry by-product hydrolysates requires a multidisciplinary and collaborative approach. Close coordination among slaughterhouses, processors, researchers, and regulatory agencies is essential for establishing standardized protocols for poultry by-product hydrolysates, covering raw material collection, process optimization, hazard control, and risk assessment. Investments in infrastructure modernization, personnel training, and digital monitoring will be essential to implement these protocols effectively. Such concerted efforts not only mitigate potential biological and chemical hazards but also support the poultry industry’s transition toward a circular, sustainable, and economically viable model of by-product utilization. Establishing a robust framework for quality and safety management will thus be fundamental for unlocking the full potential of poultry by-products as a secure and valuable source of bioactive hydrolysates for future food and health applications.

In addition, integrating consumer feedback, market trends, and evolving regulatory requirements into the process can ensure that poultry by-product hydrolysates remain safe, effective, and widely accepted, further enhancing the long-term sustainability and commercial viability of the poultry by-product industry. Looking ahead, scaling up the production of poultry by-product hydrolysates will increasingly rely on integrated, end-to-end safety and quality management systems. Advanced approaches or predictive models, such as predictive microbiology, in-line spectroscopic analysis, and machine-learning-based quality forecasting models, can help processors detect deviations in real time and maintain batch-to-batch consistency (Liao et al., 2023; Hollebrands et al., 2025). Establishing centralized databases for poultry by-product hydrolysates, including reference values for microbial loads, chemical residues, oxidation markers, and functional peptide profiles, would further support risk assessment and regulatory harmonization across the industry. Future development will also depend on sustainable processing, such as renewable energy use, water recycling, and heat recovery, as well as cross-sector collaboration to advance green extraction, enzyme recycling, and improved packaging. In addition, cross-sector partnerships will be essential for driving innovation in green extraction technologies, enzyme recycling strategies, and biodegradable packaging for extended shelf life. Long-term consumer confidence will depend on clear communication strategies and evidence-based demonstrations of product safety, efficacy, and sustainability. Developing dedicated quality marks, such as “Clinically Evaluated Peptide Ingredient” or “Sustainably Upcycled Protein”, may also help standardize expectations and strengthen trust. As research continues to reveal the health-promoting effects of poultry-derived bioactive peptides, new opportunities will emerge in markets such as sports nutrition, elderly nutrition, metabolic health, and personalized functional foods. These efforts, taken together, will be key to establishing poultry by-product hydrolysates as safe, reliable, and globally competitive ingredients for future food and health applications.

## Limitation of government regulations

### Current regulatory constraints on poultry by-products

In the European Union (EU), regulatory frameworks strictly prohibit the entry of animal by-products into the human food chain due to persistent safety concerns, contamination risks, and public health considerations that remain unaddressed or inadequately mitigated. Under EC Regulation No. 1069/2009 and its implementing Regulation (EC) No. 1774/2002, chicken by-products are categorized into three classes based on risk level and permissible end use (European Union, 2009). Category 1 materials, deemed high-risk, are prohibited from any reuse due to potential hazards, including transmissible spongiform encephalopathies, bacterial contamination, and chemical residues, such as heavy metals or veterinary drugs. Category 2 by-products are restricted to non-food applications, such as technical processing, energy recovery, composting, or incineration, ensuring they remain entirely outside the food chain. Category 3 by-products, derived from healthy animals slaughtered for human consumption, can be used for a limited number of applications, including livestock feed, pet food, aquaculture feed, cosmetics, compost, and biogas production (Lasekan et al., 2013; European Union, 2009). The implementation of these regulations reflects the EU’s precautionary principle approach to food safety, which was strongly reinforced in the aftermath of the bovine spongiform encephalopathy (BSE) crisis. That event reshaped regulatory practices for animal-derived materials, emphasizing the necessity of traceability, risk assessment, and monitoring throughout all stages of slaughter, by-product collection, handling, and processing. Furthermore, the stringent rules also aim to protect consumer confidence and maintain public trust in the safety of animal-derived products, which is crucial for both domestic and international food markets. Despite these regulations being effective for mitigating high-risk scenarios, they simultaneously restrict innovation and the potential valorization of low-risk by-products for high-value applications, creating a regulatory bottleneck for functional food and nutraceutical development. In addition, limited flexibility in permitting novel processing technologies, such as enzymatic hydrolysis or fermentation, hinders industry efforts to explore more sustainable, value-added uses of by-products while maintaining strict safety compliance.

### Technological advances enabling potential reassessment

Despite strict prohibitions, recent advances in poultry-specific proteomics and biotechnology have created new opportunities to reassess the controlled use of poultry processing by-products within scientifically validated safety frameworks. High-resolution mass spectrometry, high-performance liquid chromatography (HPLC), nuclear magnetic resonance (NMR), and next-generation proteomics now enable precise characterization of the poultry peptidome, allowing identification of bioactive peptide sequences, such as antioxidant and antihypertensive peptides, and screening for potential hazards, including microbial contamination, chemical residues, or heavy metals (Cassidy et al., 2021; Tamara et al., 2022; Aendo et al., 2024; Lešić et al., 2025). These analytical tools provide a stronger basis for distinguishing unsafe poultry waste from functional peptide fractions with potential nutraceutical value. At the same time, *in vitro* cell-based models and *in vivo* evaluations remain essential to confirm whether these poultry-derived peptides retain their bioactivity after digestion and absorption. It also supports risk-based regulatory reassessment. Regulatory reconsideration, however, must still depend on rigorous risk management, including monitoring of veterinary drug residues and heavy metals that may accumulate in poultry offal and organ tissues. In addition, the safety and functionality of poultry by-product hydrolysates are strongly influenced by hydrolysis conditions such as enzyme-to-substrate ratio and incubation time, which determine peptide profiles and molecular weight distribution. Real-time control of pH and temperature during the hydrolysis of poultry blood, viscera, or other by-products can help prevent the formation of biogenic amines and lipid deterioration. Combined with digital process control and automated sampling, these measures can improve batch consistency, traceability, and safety, thereby supporting the development of poultry by-product hydrolysates for human nutraceutical applications.

### The pet food industry as a testing platform

The pet food industry provides a pragmatic testing ground for the use of poultry by-product hydrolysates, bridging the gap between regulatory restrictions and safe commercialization (Vasconcellos et al., 2024). Recent studies have demonstrated that chicken organ hydrolysates, including liver and heart, can serve as primary protein sources in extruded diets, offering benefits such as reduced systemic inflammation, decreased oxidative stress, and improved gut health in healthy adult dogs (Hsu et al., 2024a & b). The pet food sector, supported by robust quality assurance systems, traceability mechanisms, and regulatory oversight, provides an ideal platform for validating the safe and effective use of animal by-products. It is therefore particularly suitable for assessing the production processes, safety, and functional value of by-product derived ingredients before their potential application in the human food sector. Developing a standardized regulatory framework for poultry by-product hydrolysates in pet nutrition would include detailed definitions, testing criteria, labeling protocols, and risk-based inspection systems aligned with principles such as the Hazard Analysis and Critical Control Point (HACCP) framework. Additionally, advanced digital tools, such as blockchain-based traceability systems, can document every stage of by-product handling, from slaughterhouse collection to processing and packaging, ensuring immutable verification of hygiene practices and process conditions. By providing a controlled environment for validation, the pet food industry can facilitate regulatory confidence, promote international trade through transparent verification, and establish a scientific basis for assessing safety, bioactivity, and functional properties of hydrolysate-derived peptides. This approach also allows incremental risk assessment, enabling regulators to gradually expand permissible uses in higher-value applications while maintaining strict consumer protection standards. Furthermore, lessons learned from pet food applications can inform public health communication strategies, helping consumers better understand the safety, nutritional value, and functional potential of by-products in food systems.

### Global regulatory diversity and challenges for harmonization

Globally, significant regulatory heterogeneity poses challenges for the safe and standardized use of poultry by-products and hydrolysates. While the EU enforces some of the world’s most stringent animal by-product standards, other regions, including Asia, South America, and parts of North America, adopt more flexible regulatory criteria that balance local economic realities, waste management capacities, and food security needs. For instance, in countries such as Brazil, China, and other Asian nations, poultry by-products are often repurposed for animal feed or fertilizer production under nationally defined hygiene criteria. Such regional discrepancies underscore the need for internationally harmonized standards that accommodate local practices while maintaining core safety principles. International organizations such as the Food and Agriculture Organization (FAO), the World Health Organization (WHO), and the World Organization for Animal Health (OIE) could play pivotal roles in developing guidance documents, global monitoring frameworks, and information-exchange platforms to support the safe use of by-products. Cross-border trade further complicates regulatory consistency, as variations in slaughtering, processing, and storage conditions may introduce heterogeneity in product quality and safety. Effective import and export oversight requires robust inspection, certification, and mutual recognition systems to ensure compliance with safety standards. At the same time, tailored risk assessment methodologies are needed to evaluate potential allergenicity, toxicology, and nutritional benefits. Collectively, the integration of advanced scientific techniques, digital traceability systems, and international harmonization strategies provides a pathway for the safe, sustainable, and value-added utilization of poultry by-products. These efforts are essential not only for consumer protection but also for fostering innovation, promoting the development of functional ingredients, and enhancing circular economy practices in global food systems. Furthermore, lessons learned from pet food applications can inform public health communication strategies, helping consumers better understand the safety, nutritional value, and functional potential of by-products in food systems.

## Bridging the gap between lab-scale research and commercialization

### Transition from laboratory-scale experiments to industrial production

Laboratory-scale investigations provide invaluable insights into the bioactivity, compositional profiles, and mechanistic pathways of hydrolysates derived from agricultural by-products, particularly those sourced from poultry. Nevertheless, translating these findings into industrial-scale operations poses considerable challenges. Laboratory experiments are conducted under highly controlled conditions, utilizing small batch sizes, high-purity reagents, and tightly regulated temperature, pH, and mixing conditions. These controlled environments rarely replicate the operational complexities of industrial production, which involves large volumes of heterogeneous raw materials, variable enzymatic performance, and fluctuations in environmental conditions. Consequently, scaling up hydrolysate production often reveals issues with process efficiency, enzyme stability, energy consumption, yield optimization, and the reproducibility of bioactive properties. Variability in raw material composition, such as differences in protein, lipid, and moisture content across batches, further complicates efforts to maintain product uniformity. To address these challenges, the development of standardized, scalable, and economically viable production protocols is imperative. Such protocols must ensure that the bioactive properties of hydrolysates are preserved while simultaneously achieving operational efficiency, cost-effectiveness, and regulatory compliance (Chou et al., 2014; Yang et al., 2019). Previous studies exemplify successful laboratory-to-industrial translation by precisely controlling enzyme selection, substrate-to-enzyme ratios, and hydrolysis duration, thereby achieving a balance between targeted biofunctional activity and production efficiency. Moreover, securing intellectual property rights through patent applications not only protects innovation but also facilitates technology transfer and provides strategic advantages in commercialization (Chen et al., 2018 & 2019). Additionally, integrating process modeling, real-time monitoring, and predictive analytics can further support scale-up efforts, enabling producers to anticipate bottlenecks, optimize energy and enzyme utilization, and maintain consistent bioactivity across industrial batches. Beyond technical optimization, economic analyses and life-cycle cost evaluations are also crucial to ensure that industrial-scale production is financially sustainable, particularly when aiming to produce high-value functional ingredients from low-cost by-products.

### Formulation, sensory optimization, and functional validation

Beyond industrial-scale production, formulating hydrolysates into consumer-ready functional foods or nutraceuticals requires overcoming significant sensory, stability, and bioactivity challenges. Hydrolysates often exhibit inherent bitterness, off-flavors, or undesirable textures, which may reduce consumer acceptance (Liu et al., 2022). Innovative formulation strategies are therefore critical to improve palatability without compromising the biofunctional efficacy of peptides. Such strategies include blending hydrolysates with complementary bioactive compounds, using natural flavor enhancers or masking agents, and encapsulating bioactive peptides to reduce sensory impact and improve gastrointestinal stability. Moreover, the stability of peptides under processing, storage, and simulated digestive conditions must be rigorously verified to ensure consistent physiological activity in the final product. Health claims associated with these hydrolysates, such as hepatoprotective, antioxidant, or antihypertensive activities, must be substantiated through mechanistic studies, preclinical trials, and, where feasible, human clinical validation. For instance, collaborative efforts between academic institutions and industry have successfully formulated chicken-liver hydrolysate supplements with verified antioxidant and hepatoprotective effects, demonstrating the feasibility of translating mechanistic insights into marketable products (Lin et al., 2023, 2024b, & 2026; Wu et al., 2024). Furthermore, the development of standardized analytical methods and bioactivity assays enables reproducible quality assessment and supports regulatory submissions. Such rigorous validation not only enhances product credibility and regulatory compliance but also strengthens consumer trust in functional foods and nutraceuticals. Additionally, incorporating sensory panels, consumer testing, and shelf-life studies ensures that products meet both quality and market-acceptance criteria, thereby facilitating successful commercialization.

### Quality assurance, safety, and supply chain traceability

Ensuring the safety, consistency, and traceability of hydrolysates throughout the production and supply chain is fundamental for commercial success and regulatory compliance. Implementation of internationally recognized standards, such as ISO 22000, HACCP, and GMP, provides a structured framework for harmonizing production practices, mitigating risks, and ensuring product safety. Advanced digital monitoring technologies, including real-time sensor networks, automated environmental control, and blockchain-based traceability systems, can enhance transparency, verify each stage of production, and provide immutable records of compliance with hygiene and processing standards. The adoption of environmental sustainability metrics, such as life-cycle assessment (LCA), further quantifies the ecological benefits of converting poultry by-products into high-value hydrolysates, thereby aligning production practices with global circular-economy goals. These measures, taken together, ensure that hydrolysates retain their nutritional and functional properties while minimizing environmental impact. Furthermore, integrated monitoring of raw material quality, including microbial load, heavy metals, veterinary drug residues, and lipid oxidation indices, serves as a critical safeguard against safety risks. By establishing a comprehensive quality assurance framework, producers can deliver consistent, safe, and functionally effective hydrolysates, thereby facilitating market access and international trade. Continuous data collection and integration across the supply chain also allow predictive maintenance, early detection of deviations, and proactive risk mitigation, which are essential for both product quality and consumer protection.

### Collaborative strategies for bridging research and market application

Bridging the gap between laboratory discovery and commercial application necessitates close collaboration among academia, industry, and regulatory agencies. Academic research provides fundamental knowledge of peptide composition, bioactivity, and mechanism of action, whereas industry contributes expertise in process optimization, pilot-scale validation, and market strategy. Joint research centers, innovation hubs, and technology transfer platforms can serve as effective intermediaries for pilot-scale testing, scale-up validation, and knowledge exchange. Additionally, cultivating a skilled workforce with interdisciplinary expertise spanning biochemical analysis, food engineering, regulatory compliance, and commercialization is essential for translating laboratory discoveries into viable products. Educational initiatives, specialized training programs, and interdisciplinary curricula integrating biotechnology, food science, and business management can equip the next generation of researchers with the skills needed to bridge this translational gap. Collaborative efforts should also involve continuous alignment of academic research with industrial needs, market trends, and consumer preferences to ensure that hydrolysates are not only scientifically validated but also economically viable and socially acceptable. Engaging with regulatory bodies early in product development can help streamline approval processes, enabling pilot-scale data to guide safety assessments and labeling requirements. Ultimately, integrating scientific innovation with regulatory compliance, consumer-oriented formulation, and sustainable production practices will maximize the value of poultry by-products, transforming them into high-value functional ingredients that contribute to human health, environmental sustainability, and the advancement of a circular bioeconomy. These integrative approaches further encourage cross-sector partnerships, facilitate knowledge exchange, and accelerate the commercialization of bioactive hydrolysates in both domestic and international markets. In addition, leveraging global market insights, consumer behavior analytics, and strategic branding can improve market penetration and acceptance, ensuring that scientifically validated hydrolysates achieve both commercial success and broad health impact.

## Conclusion

In recent years, research on hydrolysates derived from poultry edible by-products has made remarkable progress, driven by the rapid expansion of the global poultry industry, increasing interest in circular agriculture, and substantial advancements in analytical, proteomic, and biotechnological methodologies. These developments have created unprecedented opportunities for valorizing underutilized by-products into high-value functional ingredients with potential applications in human nutrition, nutraceuticals, and animal feed. Despite these advancements, the practical application of poultry by-product hydrolysates remains challenging. Key obstacles include navigating complex regulatory frameworks, ensuring consistent raw material quality, and maintaining both safety and functional efficacy throughout production, processing, and storage. Furthermore, the variability in raw material composition and the susceptibility of bioactive peptides to degradation pose additional constraints on industrial scalability and product standardization. Bridging the gap between laboratory-scale research and large-scale commercial production remains a critical step in fully realizing the potential of these bioactive compounds. Laboratory studies have provided detailed insights into the biochemical composition, peptide sequences, and physiological benefits of hydrolysates under highly controlled experimental conditions. However, translating these findings into industrial-scale operations requires developing standardized, reproducible, and cost-effective processing protocols. Factors such as enzyme selection, enzyme-to-substrate ratios, hydrolysis conditions, and downstream purification methods must be optimized to ensure process efficiency, high yield, and preservation of bioactivity. Equally important are rigorous quality control measures and safety frameworks to prevent microbial contamination, oxidative damage, and functional loss. At the same time, advanced monitoring systems and traceability platforms are essential for maintaining regulatory compliance and fostering consumer confidence. Close collaboration and negotiation among industry leaders, academic researchers, and governmental bodies are essential to move forward. Taken together, these groups can work toward establishing sustainable practices and developing innovative management and production models based on rigorous scientific research. Such collaboration also provides opportunities to implement new processing technologies, strengthen safety assurance systems, and refine regulatory frameworks that support the responsible utilization of poultry by-products while aligning with environmental sustainability goals. Given the current regulatory environment and the structure of the poultry industry, the pet food sector represents an ideal starting point for large-scale application and validation. Successful adoption in this field could serve as a valuable model for expanding the use of hydrolysates into other markets, including human nutrition. Looking forward, continued advancement in analytical and characterization techniques, coupled with the adoption of internationally recognized quality standards and progress toward global regulatory harmonization, will be pivotal in unlocking the full economic, nutritional, and environmental potential of poultry by-products. By integrating technological innovation, rigorous regulatory frameworks, and sustainable production practices, stakeholders can transform materials traditionally considered waste into high-value, biofunctional resources. These efforts not only enhance food system resilience and economic development but also promote environmental sustainability, resource efficiency, and the global transition toward circular agricultural systems.

## CRediT authorship contribution statement

**Yi-Hsieng S. Wu:** Writing – original draft, Validation, Data curation. **Yi-Ling Lin:** Writing – original draft, Validation, Data curation. **Yi-Chen Chen:** Writing – review & editing, Validation, Supervision, Funding acquisition, Data curation, Conceptualization.

## Disclosures

The authors declare that they have no known competing financial interests or personal relationships that could have appeared to influence the work reported in this paper.
